# A submicron forest-like silicon surface promotes bone regeneration by regulating macrophage polarization

**DOI:** 10.3389/fbioe.2024.1356158

**Published:** 2024-04-19

**Authors:** Guo Sun, Tianyu Shu, Shaoyang Ma, Meng Li, Zhiguo Qu, Ang Li

**Affiliations:** ^1^ Key Laboratory of Shaanxi Province for Craniofacial Precision Medicine Research, College of Stomatology, Xi’an Jiaotong University, Xi’an, China; ^2^ MOE Key Laboratory of Thermo-Fluid Science and Engineering, School of Energy and Power Engineering, Xi’an Jiaotong University, Xi’an, China

**Keywords:** silicon, submicron, surface topography, bone regeneration, macrophage polarization

## Abstract

**Introduction:** Silicon is a major trace element in humans and a prospective supporting biomaterial to bone regeneration. Submicron silicon pillars, as a representative surface topography of silicon-based biomaterials, can regulate macrophage and osteoblastic cell responses. However, the design of submicron silicon pillars for promoting bone regeneration still needs to be optimized. In this study, we proposed a submicron forest-like (Fore) silicon surface (Fore) based on photoetching. The smooth (Smo) silicon surface and photoetched regular (Regu) silicon pillar surface were used for comparison in the bone regeneration evaluation.

**Methods:** Surface parameters were investigated using a field emission scanning electron microscope, atomic force microscope, and contact angle instrument. The regulatory effect of macrophage polarization and succedent osteogenesis was studied using Raw264.7, MC3T3-E1, and rBMSCs. Finally, a mouse calvarial defect model was used for evaluating the promoting effect of bone regeneration on the three surfaces.

**Results:** The results showed that the Fore surface can increase the expression of M2-polarized markers (CD163 and CD206) and decrease the expression of inflammatory cytokines, including interleukin-6 (IL-6) and tumor necrosis factor alpha (TNF-α). Fore surface can promote the osteogenesis in MC3T3-E1 cells and osteoblastic differentiation of rBMSCs. Furthermore, the volume fraction of new bone and the thickness of trabeculae on the Fore surface were significantly increased, and the expression of RANKL was downregulated. In summary, the upregulation of macrophage M2 polarization on the Fore surface contributed to enhanced osteogenesis *in vitro* and accelerated bone regeneration *in vivo*.

**Discussion:** This study strengthens our understanding of the topographic design for developing future silicon-based biomaterials.

## 1 Introduction

The highly efficient restoration of a bone defect is a major clinical concern. The reason is that human bone tissue often shows limited regenerative capacity and requires proper external intervention for regeneration (Bai et al., 2018; Wang X. et al., 2018). Silicon is a major trace element in humans (Jugdaohsingh et al., 2013; Martin, 2013). Increased dietary silicon intake facilitates human skeletal health (Jugdaohsingh et al., 2004). Orthosilicic acid, biosilica, and silica nanoparticles can stimulate type I collagen synthesis and osteoblast differentiation in human osteoblast-like cells (Sun et al., 2017; Sun et al., 2018; Friguglietti et al., 2020). Silicon nitride surfaces have also shown osteogenic/antibacterial dual properties (Bock et al., 2017; Ishikawa et al., 2017; Du et al., 2022). Therefore, silicon shows some potential in stimulating bone regeneration. To develop new silicon-based biomaterials for bone regeneration, the optimized design of bioactive silicon surfaces should be completely elaborated.

The surface topography of biomaterials plays a vital role in osteogenesis (Bosshardt et al., 2017). Installed biomaterials can trigger innate immune responses and recruit macrophages (Trindade et al., 2016). To establish a suitable microenvironment for bone regeneration, the M2-polarized macrophages are necessary with their anti-inflammatory and pro-healing potential to inhibit the innate immune response (Chen et al., 2016; Zhu G. et al., 2021; Toita et al., 2022). It has been proven that proper surface topography of biomaterials can promote the M2 polarization of macrophages to enhance bone regeneration (Hotchkiss et al., 2016; Abaricia et al., 2020; Zhu Y. Z. et al., 2021). In addition, the surface topography of biomaterials can directly influence osteogenesis. That is, the material surface that mimics natural bone topography can promote protein adsorption, mesenchymal stem cell osteogenic differentiation, and trabecular bone ingrowth (Boyan et al., 2017; Shah et al., 2019; Berger et al., 2022). Thus, it is crucial to clarify the regulatory effect of silicon-based biomaterials on macrophage polarization and succedent osteogenesis.

Recently, it was shown that lithography technology shows potential in precisely tailoring the surface topography of biomaterials (Kooy et al., 2014; Brown et al., 2023). Therefore, the surface submicron pillar has been a potential pro-osteogenesis design. Submicron pillars can modulate cell behavior such as adhesion and migration (Nouri-Goushki et al., 2020; Angeloni et al., 2023). Lithographically formed silicon nanorods promote the osteopontin expression in pre-osteoblasts, which reveals its pro-osteogenesis effect (Ganjian et al., 2022). In addition, the density and height of submicron pillars have shown an immunomodulating effect in regulating M1/M2 polarization of macrophages (Nouri-Goushki et al., 2021). Specific submicron pillars (500 nm in diameter and 2 μm in height) can stimulate macrophage differentiation into osteoclasts (Akasaka et al., 2022). Notably, the submicron silicon pillars in existing studies are dominantly regular. However, the submicron features of human bone (such as lamellae, osteocytes, and the extrafibrillar matrix) are naturally irregular (Shah et al., 2019). The effect of pillar morphology should be considered further when evaluating the biological response of submicron silicon pillars.

**FIGURE 1:**
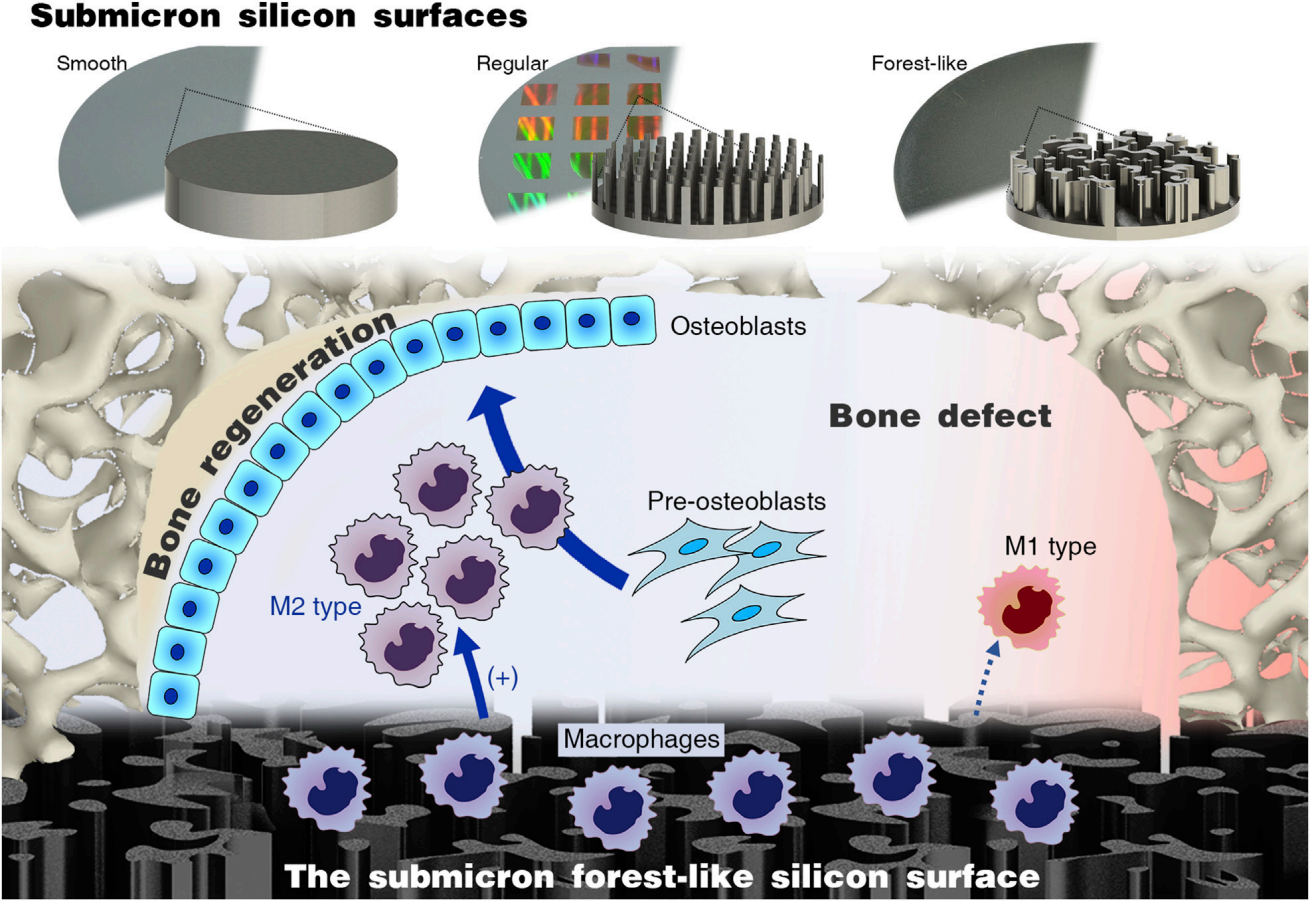
Submicron forest-like (Fore) silicon surface promotes M2-type macrophage polarization for bone generation.

Therefore, this study proposes a submicron forest-like (Fore) silicon surface for promoting bone regeneration and compares it with common submicron silicon columns and the smooth (Smo) silicon surface (Figure 1). We investigate the macrophage response and subsequent pro-osteogenesis effect. The *in vivo* bone regeneration is evaluated in the mouse calvarial defect model. The result will enhance the experimental basis for future silicon-based biomaterial designs.

## 2 Materials and methods

### 2.1 Sample preparation and characterization

The Smo, regular (Regu), and Fore submicron silicon surfaces were manufactured by Suzhou Research Materials Microtech Co., Ltd. (China). The Smo surface was a 4,000-grit polished silicon wafer. The Regu surface contained repeating photoetched columns (diameter: 400–500 nm; height: 1,000 nm). The Fore surface contained plasma-treated irregular (diameter: 250–500 nm; height: 800–1,000 nm) pillars. The surface topography was characterized using a field emission scanning electronic microscope (FE-SEM; Hitachi S-4800, Japan). Surface roughness parameters (Sa, Sq, and Sz) were detected using an atomic force microscope (AFM; 5500, Agilent, United States) in a 2.5 mm × 2.5-mm area. The surface chemical composition was analyzed using an energy-dispersive X-ray spectrometer (EDS; EDAX, United States). Surface wettability with distilled water in the air was detected using an optical contact angle meter (DSA100, Kruss, Germany). In addition, a solution containing 1% bovine serum albumin (BSA; Sigma-Aldrich, United States) was prepared, and the protein adsorption capacity of the surface of the three samples was measured using MicroBCA assay.

### 2.2 Macrophage polarization

Raw264.7 cells were seeded on the surface of each sample (1×10^4^ cells/well). After 1, 3, and 9 days, macrophages were fixed with the Gluta fixative, dehydrated with graded ethanol, ion sputtered, and then observed using the FE-SEM (Hitachi S-4800, Japan). For each group of samples, 5 fields of view were selected at 3, 6, 9, and 12 o’clock in the center and around the samples. The spread area and longest diameter of the macrophages were measured using ImageJ software. In addition, F-actin was stained using phalloidin-Alexa Fluor 488 (1:1,000; C2201S, Beyotime, China) and incubated for 30 min, followed by incubation in mounting medium with DAPI (ab104139, Abcam, United States) and was visualized using the Olympus FV3000 confocal microscope. The cell proliferation activity of the three groups of samples was detected using the CCK-8 method.

After 7 days, the expression of M1 markers (*Mhc2*, *Inos*, and *Il-6*) and M2 markers (*Cd163* and *Cd206*) was evaluated using qRT-PCR. Primer sequences are shown in Supplementary Table S1. The TNF-α level in the cell culture was detected using an ELISA kit (EK0527, Boster, China). Meanwhile, the expression of M1 markers (iNOS and CD86) and M2 markers (CD163, CD206, and Arg-1) was evaluated using Western blot. Antibodies and dilutions included anti-CD86 (1:200; ab112490, Abcam, United States), anti-iNOS (1:1,000; ab178945, Abcam, United States), anti-CD206 (1:1,000; ab64693, Abcam, United States), anti-CD163 (1:1,000; ab182422, Abcam, United States), and anti-ARG1 (1:5,000; ab233548, Abcam, United States). The anti-β-actin antibody (1:5,000, ab6276, Abcam, United States) was used for quantifying the loading amount of the sample. In addition, the expression of M2 polarization markers (CD206 and CD163) was observed by immunofluorescence. Antibodies and dilutions included anti-CD206 (1:500; ab64693, Abcam, United States) and anti-CD163 (1:1,000; ab182422, Abcam, United States). All stained samples were visualized using the Olympus FV3000 confocal microscope.

### 2.3 Osteogenesis *in vitro*

To mimic the microenvironment of bone regeneration, the MC3T3-E1 pre-osteoblasts were cultured on the three silicon surfaces and stimulated by the supernatant from macrophages cultured on the corresponding surfaces (Figure 5A). On day 7 after stimulation, the expression of osteogenic genes (*Alpl*, *Col1a1*, and *Runx2*) was detected by qRT-PCR. The alkaline phosphatase (ALP) activity was analyzed using the alkaline phosphatase activity detection kit (Beyotime, P0321S). In addition, tetracycline solution (ML6401, Mlbio, China) was added at a ratio of 1:100 to the culture medium. At 14 days, Ob fixing solution was added after PBS washing, and DAPI staining solution was added. Given that tetracycline can selectively integrate with new borne hydroxyapatite calcium (Wang et al., 2006; Sakai et al., 2010; Sordi et al., 2021), the fluorescence intensity of tetracycline was observed under a fluorescence microscope. At 21 days, 2% Alizarin Red S Staining Solution (C0138, Beyotime, China) was used to quantify deposited mineral nodules. Then, the mineralized matrix stained with Alizarin Red was destained with 10% cetylpyridinium chloride (Sigma-Aldrich) in 10 mM sodium phosphate (pH 7.0; Sigma-Aldrich), and the calcium concentration was determined using a spectrophotometer (Thermo Fisher Scientific) at 562 nm, with a standard calcium curve in the same solution.

In addition, the supernatant of the macrophages cultured on the three silicon surfaces was also used as the stimulant for rat bone marrow mesenchymal stem cells (rBMSCs). On day 3 after stimulation, ALP staining (C3206, Beyotime, China) and the expression of osteogenic proteins (RUNX2, ALPL, and OPN) were detected. Antibodies include anti-RUNX2 (1:1,000; D1L7F, Cell Signaling Technology, United States), anti-ALPL (1:1,000; 11187-1-AP, Proteintech, China), and anti-OPN (1:1,000; 22952-1-AP, Proteintech, China). The anti-β-actin antibody (1:5,000; ab6276, Abcam, United States) was used for quantifying the loading amount of the sample.

### 2.4 Bone regeneration *in vivo*

The calvaria of mice was selected as the animal model for *in vivo* osteogenesis evaluation (Wancket, 2015). To examine the bone regeneration effect of the Smo, Regu, and Fore surfaces, 54 male mice (8 weeks old) were selected and randomly divided into three groups under the ethical approval of the Biomedical Ethics Committee of Medical College, Xi’an Jiaotong University (No. 2021-1563). A 4-mm diameter critical-sized bone defect was created in the parietal bone of each mouse using an annulated bit. Each defect was subsequently filled with Smo, Regu, or Fore samples. For sample harvesting, the animals were euthanized with a lethal dose of pentobarbital sodium. The mouse craniums were collected and fixed in 10% formaldehyde for 72 h at room temperature. The samples were scanned by micro-CT (Y.Cheetah, YXLON, Germany). Two-dimensional slices with an isotropic resolution of 19 μm were generated and used for three-dimensional reconstruction. First, we reconstructed the three-dimensional images with the whole sample, including the silicon samples and mouse craniums. Next, a threshold was applied to the images to segment the silicon from the background, and the same threshold was used for all samples. A 4.5-mm-diameter round region of interest (ROI) centered around the epicenter of the defect was analyzed. After setting a determinate threshold, the new bone volume fraction (bone volume/total volume, BV/TV) and trabecular thickness (tb.th) were obtained.

After micro-CT analysis, the craniums were decalcified in 9% formic acid, and then, the implants were removed gently from the craniums. The decalcified craniums were dehydrated in gradient ethanol, embedded in paraffin, cut into slices, and stained with H&E. The obtained sections were also dewaxed in xylene, hydrated in gradient ethanol, incubated in 0.3% hydrogen peroxide, blocked with 1% goat serum (Sigma-Aldrich, United States), and incubated with RANKL (anti-RANKL antibody, 1:200, Abcam, ab216484). Examination and analysis were performed in blind.

### 2.5 Statistical analysis

Data on biological experiments were obtained from at least three parallel experiments and presented as the mean ± standard deviation. The animals were randomly grouped before surgical treatment. Statistical differences were determined using one-way ANOVA with Tukey’s *post hoc* test.

## 3 Results

### 3.1 Surface characterization

**FIGURE 2:**
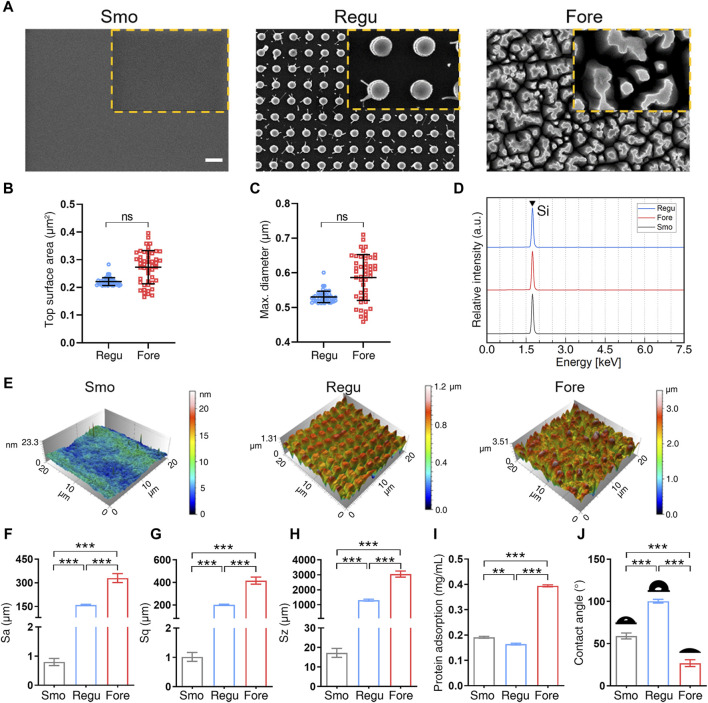
Surface characterization. **(A)** Field emission scanning electronic microscope (FE-SEM) images of the three surfaces. Scale bar: 1 μm. **(B)** Top columnar area of regular (Regu) and Fore samples. **(C)** Particle size of Regu and Fore samples. **(D)** Element component of the three surfaces. **(E)** Representative atomic force microscope (AFM) images of the three surfaces. **(F–H)** Surface roughness parameters, including Sa **(F)**, Sq **(G),** and Sz **(H)**. **(I)** Protein adsorption capacity of the three surfaces. **(J)** Contact angle with deionized water of the three surfaces. One-way ANOVA with Tukey’s *post hoc* test was used for statistical analysis. ns: no significance. *p < 0.05, **p < 0.01, and ***p < 0.001.

As shown in Figure 2A, the Regu silicon surface presented cylindrical pillars with uniform spacing, while the Fore silicon surface presented pillars with irregular shape and space. No representative submicron feature appeared on the Smo silicon surface. The average top surface area (Figure 2B) and maximum diameter (Figure 2C) of the pillars in Regu and Fore surfaces showed no statistical difference. The dominant element of the three samples was silicon (Figure 2D). Atomic force microscopy (Figure 2E) revealed that the surface roughness parameters (Sa, Sq, and Sz) of the Fore surface were increased compared to the Regu and Smo surfaces (Figures 2F–H). In addition, the Fore surface showed enhanced protein adsorption (Figure 2I) and hydrophilicity (Figure 2J).

### 3.2 Promoted macrophage M2 polarization on the Fore surface

**FIGURE 3:**
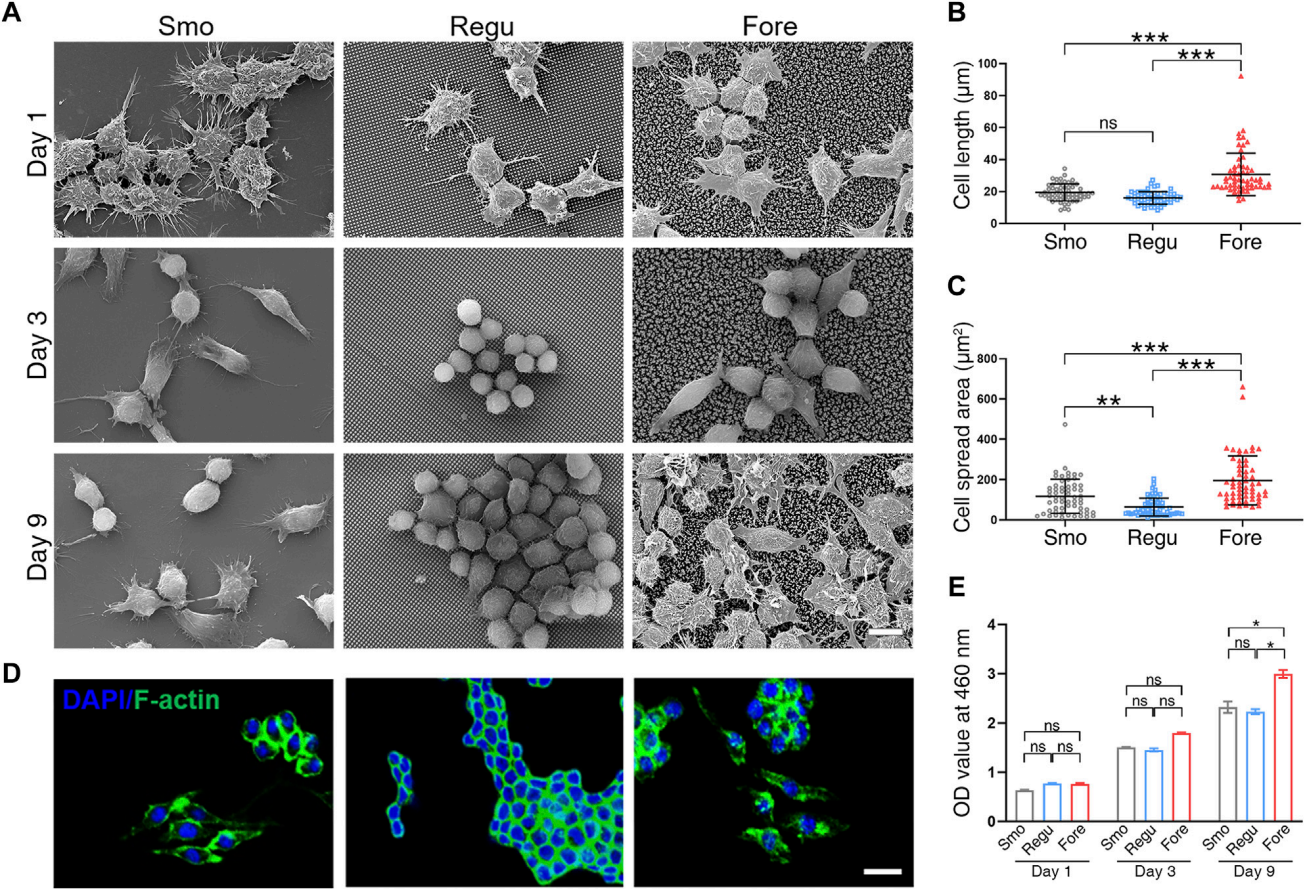
Morphological changes in Raw264.7 cells on the three surfaces. **(A)** Representative SEM images of cells on different surfaces. **(B)** Cell elongation and **(C)** cell spreading were measured. **(D)** Representative LSCM images of F-actin distribution in cells. **(E)** Proliferative activity of Raw264.7 cells on the three samples obtained by CCK-8 assay. Scale bar: 10 μm. One-way ANOVA with Tukey’s *post hoc* test was used for statistical analysis. ns: no significance. *p < 0.05, **p < 0.01, and ***p < 0.001.

The morphology of macrophages cultured on the three surfaces was observed first (Figure 3A). At 1 day after seeding, macrophages on the Smo and Fore surfaces fused. After 3 days, these macrophages on the Smo and Fore surfaces elongated and connected *via* pseudopodia. This trend was more notable after 9 days. On the other hand, macrophages on the Regu surface remained relatively round in shape, which implied the characteristics of colonization. Quantifications of the cell length (Figure 3B) and spread area (Figure 3C) showed significant differences between the three groups. Therefore, the Fore surface showed a significantly higher cell length and spread area. The observation obtained by confocal microscopy (Figure 3D) confirmed this trend. Moreover, the proliferation of macrophages cultured on the Fore surface increased 9 days after seeding (Figure 3E).

**FIGURE 4:**
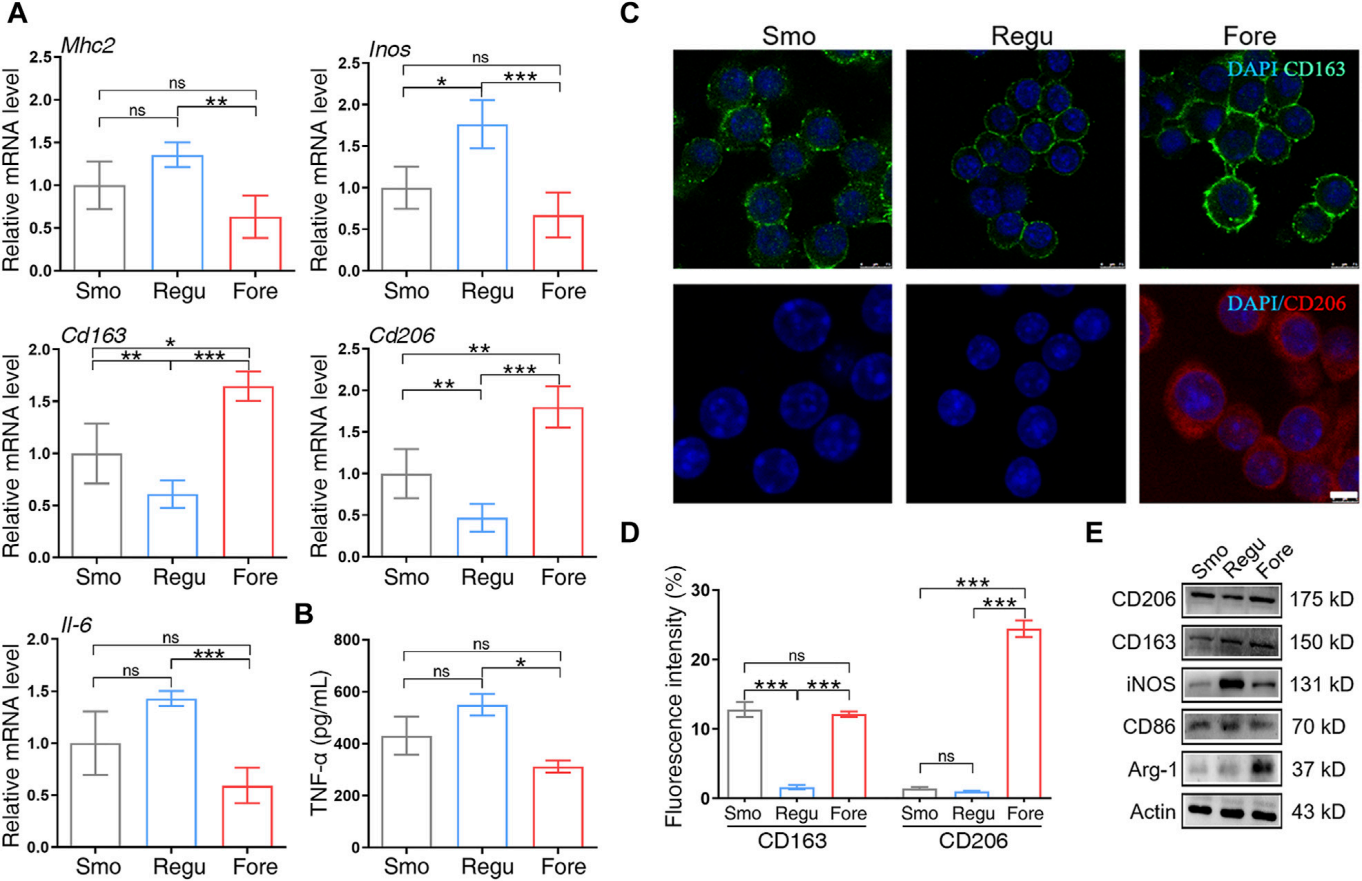
Macrophage polarization on the three surfaces. **(A)** Relative gene expression of M1 and M2 polarization markers of macrophages cultured on the three surfaces. **(B)** ELISA detection of tumor necrosis factor alpha (TNF-α) on the three surfaces. **(C, D)** Immunofluorescent staining and semiquantitative analysis for CD163 and CD206. Scale bar: 25 μm. **(E)** Protein expression of M1 and M2 polarization markers of macrophages cultured on the three surfaces. One-way ANOVA with Tukey’s *post hoc* test was used for statistical analysis. ns: no significance. *p < 0.05, **p < 0.01, and ***p < 0.001.

qRT-PCR showed that the mRNA expression level of M1 polarization markers (*Mhc2*, *Inos*, and *Il-6*) in the Fore group was significantly decreased, while the mRNA expression level of M2 polarization markers (CD163 and CD206) was significantly increased (Figure 4A). At the same time, the levels of TNF-α secreted by Raw264.7 cells on the surface of Fore samples were significantly reduced (Figure 4B). The immunofluorescence intensity of CD163 and CD206 in the Fore group was significantly enhanced, with statistical differences (Figures 4C, D). This trend was also verified using Western blot (Figure 4E) that the protein expression of M2 polarization factors (CD206, CD163, and Arg-1) on the Fore sample increased significantly, and the protein expression of M1 polarization factors (CD86 and iNOS) decreased significantly.

### 3.3 Enhanced *in vitro* osteogenesis on the Fore surface

**FIGURE 5:**
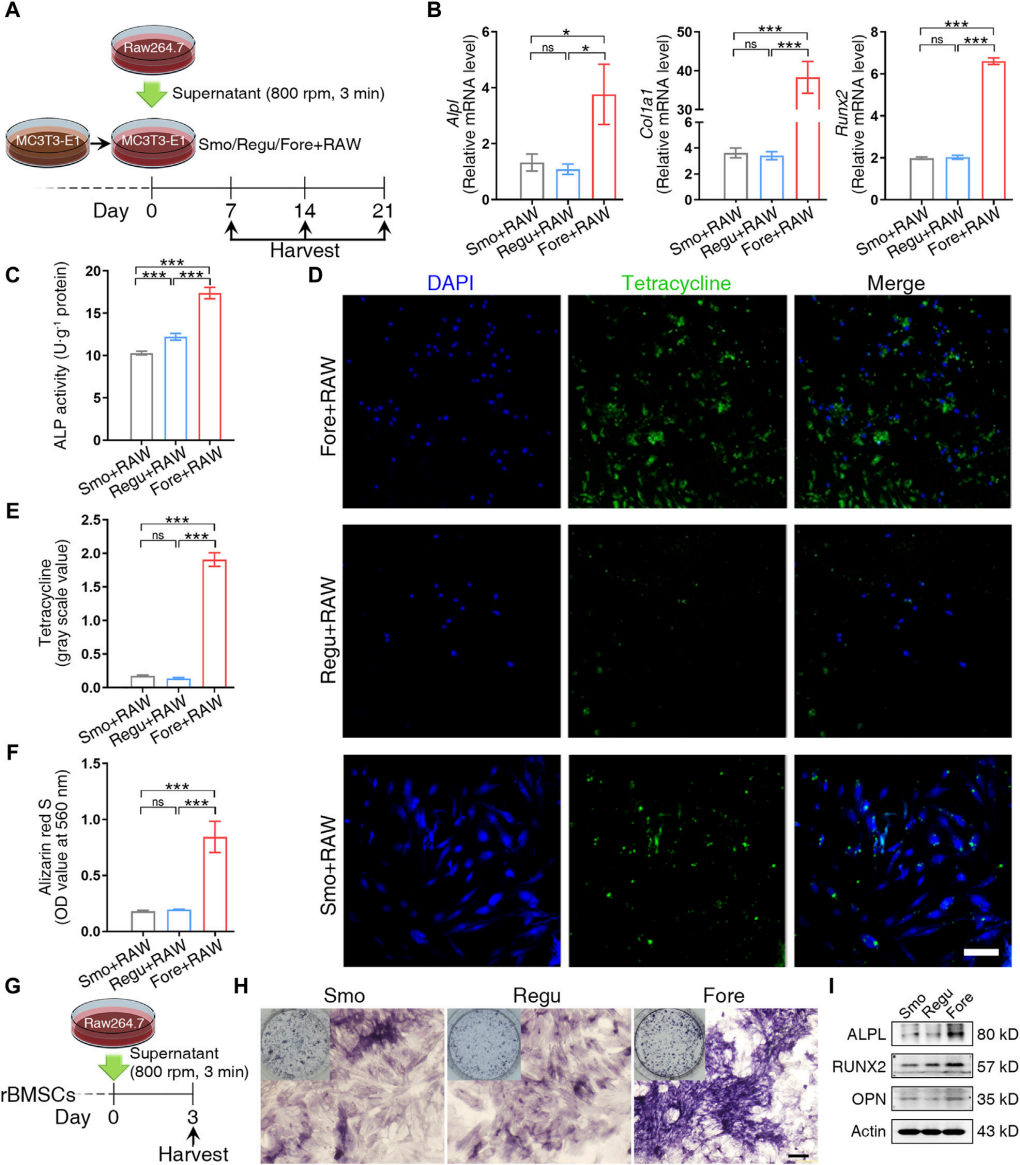
Cell osteogenic differentiation on the surface of the three samples. **(A)** Schematic diagram of MC3T3-E1 cell management. **(B)** Relative gene expression of osteogenic genes of MC3T3-E1 cells cultured on the three surfaces on day 7. **(C)** Alkaline phosphatase (ALP) activity of MC3T3-E1 cells cultured on the three surfaces on day 7. **(D, E)** Tetracycline distribution in MC3T3-E1 cells cultured on the three surfaces and semiquantitative analysis on day 14. White scale bar: 100 μm. **(F)** Semiquantification of the calcium nodule formation evaluation by Alizarin Red staining on day 21. **(G)** Schematic diagram of rat bone marrow mesenchymal stem cell (rBMSC) management. **(H)** ALP activity staining. Black scale bar: 200 μm. **(I)** Protein expression of osteogenic markers. One-way ANOVA with Tukey’s *post hoc* test was used for statistical analysis. ns: no significance. *p < 0.05, **p < 0.01, and ***p < 0.001.

After being stimulated by the supernatant from macrophages cultured on corresponding surfaces (Figure 5A), the expression of osteogenic genes (*Alpl*, *Col1a1*, and *Runx2*) of MC3T3-E1 cells in the Fore + RAW group increased significantly on day 7 (Figure 5B). At the same time, the ALP activity in the Fore + RAW group was significantly higher (Figure 5C). The promotion of osteogenesis was also observed by immunofluorescence by tetracycline staining in the Fore + RAW group at 14 days (Figure 5D, E). Similar trends were also found in the staining of Alizarin Red S at 21 days (Figure 5F and Supplementary Figure S1), with more mineral nodules in the Fore + RAW group.

In rBMSCs stimulated by the supernatant from macrophages cultured on the three silicon surfaces (Figure 5G), the alkaline phosphatase activity (Figure 5H) and the expression of RUNX2, ALPL, and OPN (Figure 5I) were increased significantly. Overall, these results proved that the immune microenvironment of the Fore surface could promote bone formation.

### 3.4 Promoted *in vivo* bone regeneration on the Fore surface

**FIGURE 6:**
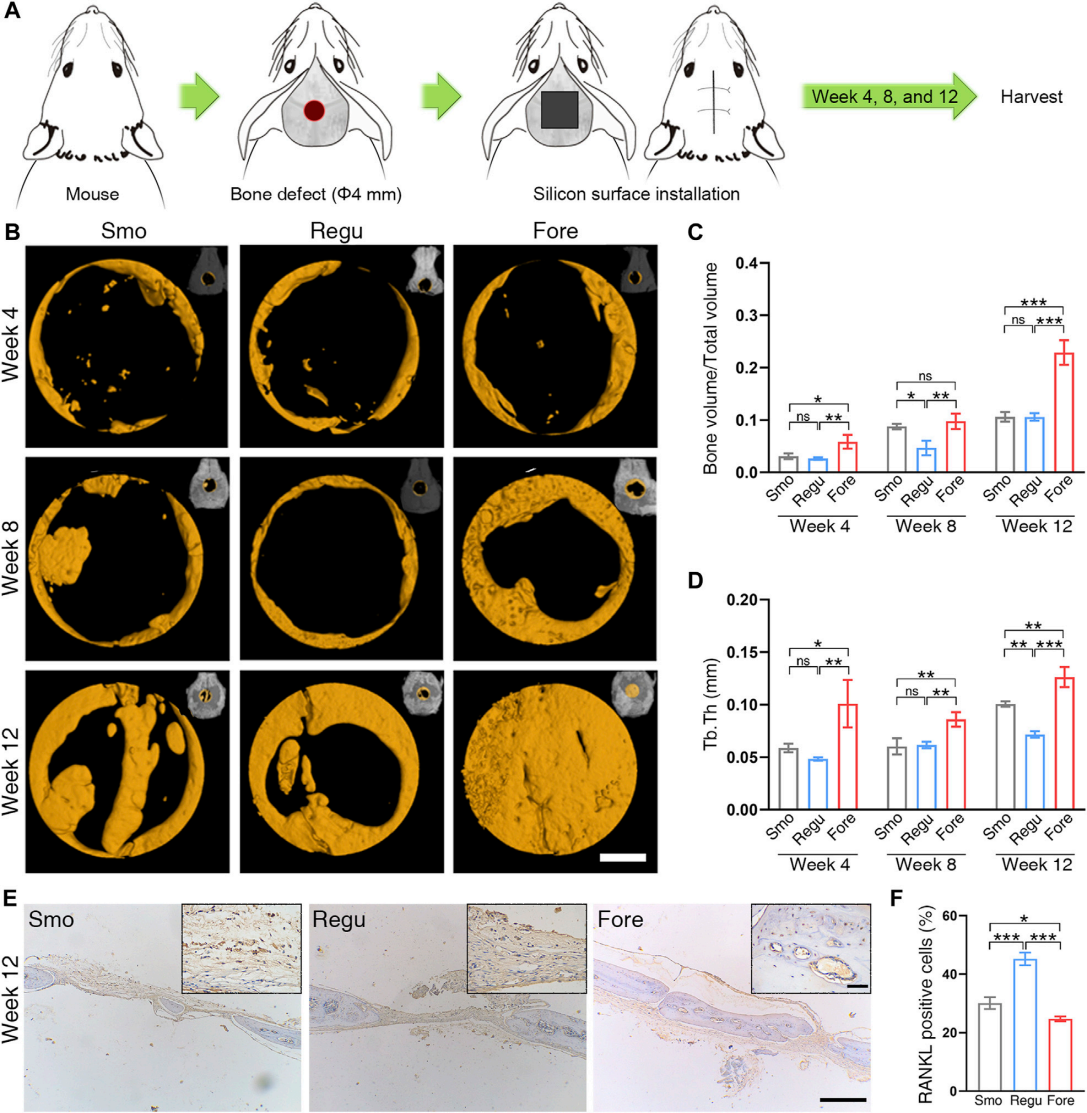
Bone regeneration of the mouse calvarial defect. **(A)** Calvarial defect model. **(B)** Bone growth in the calvarial defect at 4, 8, and 12 weeks after surgery revealed by micro-CT. White scale bar: 1.0 mm. **(C)** Quantitative analysis of bone volume/total volume (BV/TV) based on micro-CT. **(D)** Quantitative analysis of trabecular thickness (Tb.Th) based on micro-CT. **(E)** RANKL expression in the calvarial bone revealed by immunohistochemistry at 12 weeks. Long black scale bar: 500 μm. Short black scale bar: 50 μm. **(F)** Quantification of RANKL staining. One-way ANOVA with Tukey’s *post hoc* test was used for statistical analysis. ns: no significance. *p < 0.05, **p < 0.01, and ***p < 0.001.

As shown in Figure 6A, the bone regeneration was investigated with a round (Φ 4 mm) bone defect on the mouse calvaria. At 4 weeks post-implantation, a new bone began to form on the edge of the bone defect in all 3 groups, and at 8 weeks and 12 weeks, the new bone on the surface of Fore samples was significantly higher than those in Smo and Regu samples, respectively (Figures 6B–D). Histological analysis of the heart, liver, spleen, lung, and kidney at 12 weeks confirmed good biosafety of all Smo, Regu, and Fore specimens (Supplementary Figure S2). At 12 weeks, the expression level of RANKL in the new bone on the Fore surface decreased significantly (Figures 6E, F).

## 4 Discussion

In this study, a submicron forest-like silicon surface was proposed to verify whether its irregular distribution of silicon pillars on biomaterials can promote bone regeneration. The result showed enhanced macrophage M2 polarization and succedent osteogenesis *in vitro* and promoted bone defect closure *in vivo* compared with the submicron regular and smooth silicon surfaces. Therefore, the irregular forest-like submicron pillar is a potential design for developing silicon-based bone healing biomaterials.

Human bone is a complex multi-scale hierarchical structure (Reznikov et al., 2014). Therefore, varied surface topographies at the micron, submicron, and nano-scales are developed in current intrabony implants (Shah et al., 2019) and are closely related to bone regeneration. It has been well documented that the micron topography facilitates the mechanical anchorage between bone and biomaterials, while the nano-topography offers more adhesion positions for proteins at the early stage of bone regeneration (Albrektsson and Wennerberg, 2019). However, the significance of submicron topography has not been clearly defined yet. A typical submicron topography is sandblasted and acid-etched (SLA) pits on titanium implants, which is the dominant surface topography in dental implants (Smeets et al., 2016). The SLA implants have shown a less than 5% failure rate at 10 years post-implantation (Albrektsson and Wennerberg, 2019), which implies the positive effect of submicron surface topography in biomaterials on bone regeneration. In this study, the irregularly distributed Fore structure showed higher potential in stimulating the M2 polarization of macrophages and osteogenesis of pre-osteoblasts. This finding is an important supplement to the correlation with surface topography and macrophage polarization. The forest-like silicon surface shows a larger pore size and more abundant porous structure than the regular silicon pillar surface, which may explain the better anti-inflammatory activity and osteogenesis potential (Garg et al., 2013; Rustom et al., 2019). The Fore surface was more hydrophilic than the Regu surface (Figure 2J). Macrophages adhered to a hydrophilic surface tend to enhance the secretion of anti-inflammatory cytokines and M2 surface markers (Hotchkiss et al., 2016; Lv et al., 2018). In addition, it is found that the “topographical effect” plays important roles in macrophage behavior and osteogenesis (Ma et al., 2014; Wang J. et al., 2018). Sa, Sq, and Sz may influence the anti-inflammatory activity and osteogenesis potential. Overall, the result of this study echoes the current opinion that material surfaces resembling the natural bone structure are beneficial to osteogenesis (Boyan et al., 2017; Shah et al., 2019; Berger et al., 2022).

To date, titanium-based implants are holding the largest market share in tooth and bone restoration (Kaur and Singh, 2019). However, the biocompatibility of titanium is gradually being questioned due to inflammatory reactivity or metal hypersensitivity in some patients (Buettner and Valentine, 2012). Further improvement of surface biocompatibility is pivotal in developing the next generation of intrabony implants (Bandyopadhyay et al., 2023). Silicon, as a major trace element in humans (Martin, 2013), shows relatively stable *in vivo* metabolism (Jugdaohsingh et al., 2013). Wang et al. (2023) prepared silicon-deposited coatings on Ti-based implants via electron beam evaporation (Wang et al., 2023), which showed osteoinductive and immunomodulatory capacity. It was found that the burst release of Si dominated the early stages of implantation to create a favorable osteoimmunomodulatory microenvironment by the timely conversion of macrophages from an M1 to an M2 phenotype that facilitated osteogenic differentiation. In addition, the release of Si also directly activated stem cells and remodeled the extracellular matrix for late osseointegration. For instance, Francesco et al. increased the surface porosity and biological activity of the implants by embedding Si_3_N_4_ particles on the surface of PEEK implants (Boschetto et al., 2021), which showed the advantages of a low elastic modulus, improved bone integration, and promoted antibacterial effects. At the same time, a porous scaffold based on a Fe–Si alloy was manufactured by 3D printing technology, which could reduce cytotoxicity and improve mechanical stability and bone integration ability (Bondareva et al., 2022). Similar findings have been made in other implants, for example, Edward et al. found that mechanically matched silicone brain implants could potentially improve the long-term functionality and reliability of brain implants by minimizing strain and stress due to movements and swelling of the brain in both lateral and axial directions relative to the implant (E. Zhang D. H. et al., 2021). The result of this study further suggests that irregular submicron silicon surfaces are better than regular submicron silicon surfaces in bone regeneration, which is important for fabricating and optimizing future silicon-based biomaterials.

In addition, the fate of macrophages can be regulated by the surface topography of biomaterials (Hotchkiss et al., 2016; Abaricia et al., 2020; Zhu G. et al., 2021). Existing studies have confirmed that the shape and polarization of macrophages can be influenced by nanotopography of biomaterials (Ma et al., 2014; Neacsu et al., 2014; Wang J. et al., 2018). Nanotubes with a larger diameter are prone to induce M2 polarization of macrophages (Lü et al., 2015; Xu et al., 2019; Yu et al., 2021). The result of this study supports that submicron morphologies at the range of 250–500 nm can also manipulate macrophage polarization, which is an important supplement to current theories.

The control of immune responses after biomaterial implantation in the body has long been a concern in the development of medical implants (Naik et al., 2018). The increase in the number of M2-like macrophages in the damaged tissue has been suggested to be an essential event in tissue healing. Delayed polarization from the inflammatory M1 phenotype into the anti-inflammatory/healing M2 phenotype would lead to compromised stem/progenitor cell response to inhibit the functional regeneration of skin, muscle, heart, nerve, and bone (Zhang E. N. et al., 2021; Li et al., 2021). In this proof-of-concept report using bone as a model tissue, we demonstrate tissue–biomaterial integration in irregular forest-like submicron silicon-based coating with the concurrent modulation of the macrophage response and cytokine profile manifested in the M2-based phenotype. This phenotype switching is pivotal in improving tissue regeneration induced by biomaterials (Huyer et al., 2020; Schlundt et al., 2021). Osteoclasts play a key role in the process of bone regeneration, and RANKL (ligand), expressed by osteoblasts and bone marrow stromal cells, attaches to RANK receptors on mature osteoclasts and osteoclast progenitor cells and promotes their differentiation (Shah et al., 2018; Huntley et al., 2019). In addition to inducing osteoclast activity, RANKL also induces osteoclasts to attach to the bone surface and longevity (Chaparro et al., 2022). In this study, we evaluated the expression of RANKL in the new bone and found that RANKL expression decreased significantly in the Fore group while increased significantly in the Regu group, which was consistent with the results of bone regeneration.

## 5 Conclusion

In this study, an irregular forest-like submicron silicon surface was proposed as a promising design of biomaterials for bone regeneration. It demonstrated better *in vitro* and *in vivo* biological responses than the regular submicron silicon surface and smooth silicon surface, including promoting macrophage M2 polarization and facilitating bone formation. This finding enriches the research basis for silicon-based biomaterials and highlights the irregular surface design for better bone regeneration performance.

## Data Availability

The datasets presented in this study can be found in online repositories. The names of the repository/repositories and accession number(s) can be found in the article/Supplementary Material.
