# Supplementary material for: A submicron forest-like silicon surface promotes bone regeneration by regulating macrophage polarization
Source: Front Bioeng Biotechnol. 2024 Apr 19;12:1356158. doi: 10.3389/fbioe.2024.1356158 (PMC11066256; doi:10.3389/fbioe.2024.1356158)
Supplement: Supplementary file 1 [file DataSheet1.docx]

Supplementary Material


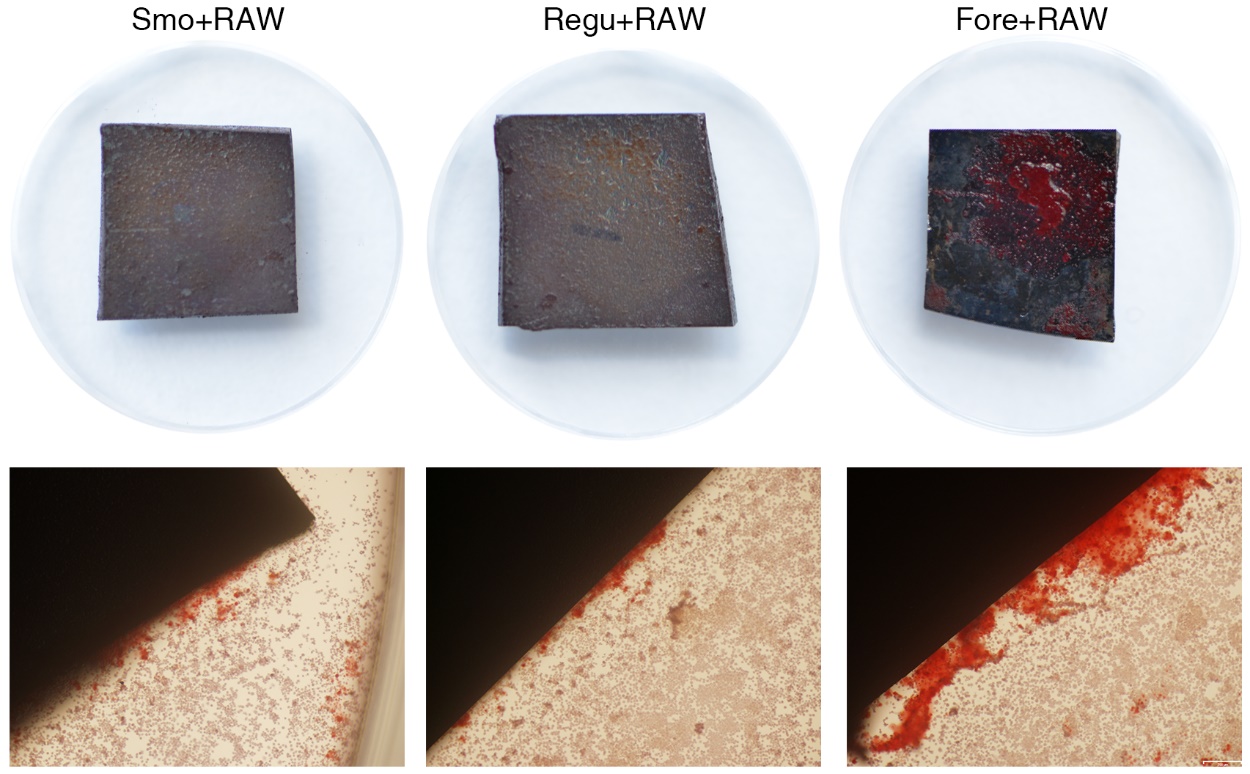


**Supplementary Figure S1.** Alizarin Red S staining of Smo, Regu, and Fore samples. Scale bar: 200 μm.


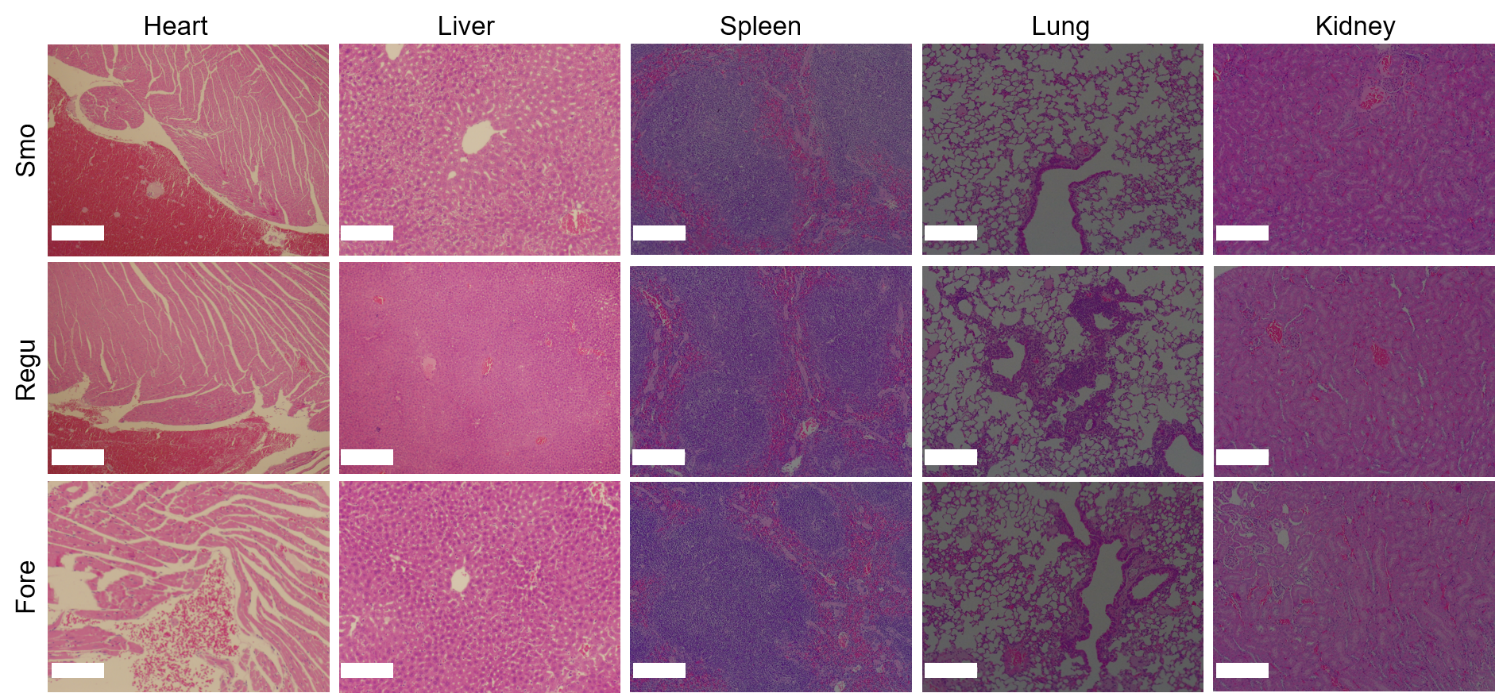


**Supplementary Figure S2.** HE stained heart, liver, spleen, lung and kidney at 12 weeks after surgery. Scale bar: 200 μm.

**Supplementary Table S1.** Primers used in qRT-PCR.

| Gene | Forward Sequence | Reverse Sequence |
| --- | --- | --- |
| *Mhc2* | GTTGAGCCTTCTGGACTGGGAAAC | CCTCTGCCGCCTGGAGTAGC |
| *Inos* | ATCTTGGAGCGAGTTGTGGATTGTC | TAGGTGAGGGCTTGGCTGAGTG |
| *Cd163* | AATCACATCATGGCACAGGTCACC | TCGTCGCTTCAGAGTCCACAGG |
| *Cd206* | CCTATGAAAATTGGGCTTACGG | CTGACAAATCCAGTTGTTGAGG |
| *Il-6* | CTTCTTGGGACTGATGCTGGTGAC | AGGTCTGTTGGGAGTGGTATCCTC |
| *Alpl* | CACGGCGTCCATGAGCAGAAC | CAGGCACAGTGGTCAAGGTTGG |
| *Col1a1* | GAAGTCAGCTGCATACACAA | GCTGATTTTTCATCATAGCC |
| *Runx2* | CCCAGCCACCTTTACCTACA | TATGGAGTGCTGCTGGTCTG |
| *β-actin* | CCCTGAAGTACCCCATTGAA | CTTTTCACGGTTGGCCTTAG |
